# Impact of a Virtual Clinic in a Paediatric Cardiology Network on Northeast Brazil

**DOI:** 10.1155/2015/504015

**Published:** 2015-07-21

**Authors:** Juliana Sousa Soares de Araújo, Adalberto Vieira Dias Filho, Renata Grigório Silva Gomes, Cláudio Teixeira Regis, Klecida Nunes Rodrigues, Nicoly Negreiros Siqueira, Fernanda Cruz de Lira Albuquerque, Felipe Alves Mourato, Sandra da Silva Mattos

**Affiliations:** ^1^Círculo do Coração, Avenida Portugal No. 163, Segundo Andar-Paissandu, 52010-010 Recife, PE, Brazil; ^2^Célula C., Avenida Portugal No. 163, Segundo Andar-Paissandu, 52010-010 Recife, PE, Brazil

## Abstract

*Introduction*. Congenital heart diseases (CHD) affect approximately 1% of live births and is an important cause of neonatal morbidity and mortality. Despite that, there is a shortage of paediatric cardiologists in Brazil, mainly in the northern and northeastern regions. In this context, the implementation of virtual outpatient clinics with the aid of different telemedicine resources may help in the care of children with heart defects. *Methods*. Patients under 18 years of age treated in virtual outpatient clinics between January 2013 and May 2014 were selected. They were divided into 2 groups: those who had and those who had not undergone a screening process for CHD in the neonatal period. Clinical and demographic characteristics were collected for further statistical analysis. *Results*. A total of 653 children and teenagers were treated in the virtual outpatient clinics. From these, 229 had undergone a neonatal screening process. Fewer abnormalities were observed on the physical examination of the screened patients. *Conclusion*. The implementation of pediatric cardiology virtual outpatient clinics can have a positive impact in the care provided to people in areas with lack of skilled professionals.

## 1. Introduction

Congenital heart diseases (CHD) affect 8 to 10 per 1000 live births [1]. They are responsible for most of the neonatal morbidity and mortality and are considered a public health issue [2, 3].

In spite of these characteristics, some countries lack trained personnel able to treat children with CHD [4]. Brazil is included among them, mainly in its northern and northeastern regions [5]. The state of Paraíba is located in the northeast region and, until recently, faced this situation.

This scenario was changed with the introduction of a paediatric cardiology network between the states of Pernambuco and Paraíba in 2012. The network established a neonatal screening program based on a focused cardiovascular physical examination, pulse oximetry, and screening echocardiogram. Nurses and neonatologists perform these exams under cardiology supervision via telemedicine when necessary [6, 7]. However, patient follow-up was also necessary and the lack of trained personnel in the area hindered the process. Thus, virtual pediatric cardiology outpatient clinics (VPCC) were created to allow local paediatricians to manage children with CHD with the online help of paediatric cardiologists.

The aim of this paper is to describe and analyse the main findings from these VPCC.

## 2. Methods

Patients less than 18 years old treated at the virtual outpatient clinics between January 2013 and May 2014 were selected. They were divided into two groups: those who were referred to the VPCC from the neonatal screening program and those who were referred to the VPCC through other processes. The virtual outpatient clinics consisted of a live meeting with a paediatrician with the remote aid of a paediatric cardiologist through the use of tablets and a communication software, WebEx, from Cisco (http://www.webex.com.br/).

The following clinical variables were analysed: general health, respiratory and cardiac auscultation, the presence of hepatomegaly, and precordium characteristics. Regarding the social variables, gender, age, and place of origin were analysed.

Data were tabulated and analysed using Microsoft Excel Spreadsheet software (version 13.0), whereas flow maps were created using TabWin software (version 3.6). Categorical variables were compared with the chi-square test using Epi Info software (version 7.1.4).

The Ethics Committee on Health and Research from Oswaldo Cruz Hospital Complex approved this paper. This research received no specific grant from any funding agency from public, commercial, or not-for-profit sectors.

## 3. Results

A total of 653 patients were analysed. There were 230 referred to the clinics from the neonatal CHD screening program and the remaining ones were referred to the clinics through other sources such as family health centers or other health care professionals. As for the year of consultation, 339 patients joined the clinics in 2013 and the remaining 314 joined in 2014. A total of 987 consultations were held, with an average of 1.5 consultations per patient. There were 606 visits in 2013 and 381 in 2014.

**Figure 1:**
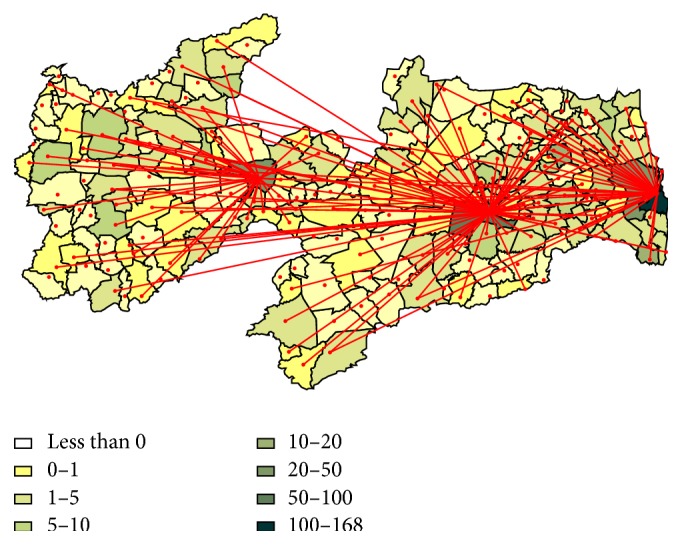
Flow map of patients followed up in the virtual clinic of paediatric cardiology.

The online outpatient clinics were established in three major centers located in three distinct regions of Paraíba.  Figure 1 depicts the patient's city of origin, the flux, and location of the three above mentioned centers.

Regarding the patients' demographics, there was a slight female predominance in gender (49.2%) with an average age of 3.04 years. Most of the patients were half-bred (68.1%), followed by whites (17.6%) and blacks (7%).  Table 1 details these data.

Roughly one-third of the patients (35.1%) were referred to the clinics from the network screening programs. No symptoms were reported in 12.3% and 10.1% were in a postoperative follow-up process.  Table 2 demonstrates the patients' clinical characteristics for both referral groups. The patients' diagnoses can be seen in Table 3.

## 4. Discussion

The lack of expert and trained health care professionals is a problem discussed worldwide [4]. Data obtained from the Ministry of Health in Brazil points to a 78.72% shortage of specialized services in paediatric cardiology in Paraíba [8].

Up to 2011, this fact hindered the patients' access to specialized services in paediatric cardiology due to the lack of doctors and other trained professionals who could only be found in large urban centers.

This context has been modified in Paraíba after the implementation of a paediatric cardiology network [9]. Its operation is based on the use of telemedicine with accessible technology, enabling the communication of several professionals throughout Paraíba with medical specialists located in the southern neighbor state (Pernambuco). Moreover, the network established a neonatal screening program for congenital heart diseases through physical examination, pulse oximetry, and screening echocardiogram [6]. However, the follow-up of the diagnosed children was also necessary.

Therefore, virtual outpatient pediatric cardiology clinics were devised. Their goal was to provide quality of care for the follow-up of patients diagnosed with heart diseases in Paraíba. As shown in Figure 1, the clinics were spread through several regions of Paraíba. Similar to what has been previously described [10], in order to optimize patients' health care in this scenario of very long distances and limitation of resources, regionalization of services was necessary. This was achieved with the use of telemedicine. The use of tablets and WebEx software made the process easier as they are relatively cheap and accessible technologies.

The neonatal screening program was capable of absorbing part of the demand for specialists due to the close online collaboration between neonatologists and pediatric cardiologists. This fact is exemplified in Table 1, which demonstrates that 35.1% of patients consulted in the clinic were referred from the screening program. On the other hand, patients who did not undergo the screening process or those who were born before the network were also included in the program. The majority of them, however, required clinical follow-up due to the type of CHD and needed management adjustments to improve their quality of life.

It is also possible to notice that when both patient groups are compared, those who underwent neonatal screening presented better clinical parameters. This is due to the early diagnosis and treatment that were made possible by the screening process in online collaboration with heart specialists leading to improved management and better quality of life for the patients.

Regarding the types of lesions observed in the clinics, there is a certain difference between the frequencies reported in the literature and in this study. For example, in this study, the most frequent CHD without cyanosis was the persistence of the ductus arteriosus and the most frequent cyanotic CHD was the Tetralogy of Fallot. Other studies show that Ventricular Septal Defect (VSD) was the most frequent CHD without cyanosis [11] and the Transposition of Great Arteries was the most frequent CHD with cyanosis [11, 12]. However, this fact may have multiple explanations such as the population of the study which was composed of many young children referred from the network's screening program as well as the methods used for its recording.

## 5. Conclusion

The implementation of the virtual outpatient clinics had a significant impact on the health of children with heart diseases in Paraíba. That was only possible due to the use of telemedicine with cheap technology as it allowed for the regionalization of services under remote specialized professional support. Its expansion to other specialties may present similar results.

## Figures and Tables

**Table 1:** Profile of patients served in the RCP virtual clinic.

Patients' profile	Total	
	(*n* = 653)	%
Sex		
Female	321	49,2%
Male	309	47,3%
Uninformed	23	3,5%
Age (in years)		
Minimum	0,02	
Maximum	17,46	
Mean	3,04 (±3,77)	
Ethnicity		
White	115	17,6%
Black	46	7,0%
Half-bred	445	68,1%
Uninformed	46	7,0%

**Table 2:** Comparison of clinical examination among patients according to mode of access to RCP virtual clinic.

	Origin				
	Triage program		Other forms		Sig.
	(*n* = 230)	%	(*n* = 423)	%	
HDA (first medical consultation)					<0,001^*∗*^
Asymptomatic	0	0,0%	80	18,9%	
Surgical evaluation	0	0,0%	11	2,6%	
Neonatal Echo follow-up	229	99,6%	0	0,0%	
Postoperative follow-up	0	0,0%	66	15,6%	
Others	1	0,4%	266	62,9%	
Medical management (last medical consultation)					0,260^*∗*^
Hospital admission	38	16,5%	52	12,3%	
Hospital discharge	3	1,3%	9	2,1%	
Follow-up	189	82,2%	362	85,6%	
General state (first medical consultation)					<0,001^*∗*^
Good	224	97,4%	377	89,1%	
Decayed	0	0,0%	5	1,2%	
Regular	6	2,6%	41	9,7%	
General state (last medical consultation)					0,014^*∗*^
Good	223	97,0%	385	91,0%	
Decayed	0	0,0%	3	0,7%	
Regular	7	3,0%	35	8,3%	
Skin (last medical consultation)					0,244^*∗*^
Cyanotic	3	1,3%	15	3,5%	
Colored	213	92,6%	384	90,8%	
Pale	14	6,1%	24	5,7%	
Respiratory pattern (last medical consultation)					0,047^*∗*^
Dyspnea	17	7,4%	15	3,5%	
Eupneic	213	92,6%	408	96,5%	
Pulmonary auscultation (last medical consultation)					0,504^*∗∗*^
Anormal	2	0,9%	7	1,7%	
Normal	228	99,1%	416	98,3%	
Abdomen (last medical consultation)					0,153^*∗*^
Hepatomegaly	11	4,8%	9	2,1%	
Normal	218	94,8%	413	97,6%	
Others	1	0,4%	1	0,2%	
Perfusion (last medical consultation)					0,167^*∗*^
Normal	230	100,0%	418	98,8%	
Compromised	0	0,0%	5	1,2%	
Peripheral pulses (last medical consultation)					1,000^*∗*^
Anormal	2	0,9%	4	0,9%	
Normal	228	99,1%	419	99,1%	
Murmur (first medical consultation)					0,371^*∗*^
Absent	125	54,3%	213	50,4%	
Present	105	45,7%	210	49,6%	
Continuous	4	3,8%	8	3,8%	
Diastolic	0	0,0%	2	1,0%	
Systolic	101	96,2%	200	95,2%	

^*∗*^Chi-square test.

^*∗∗*^Fisher's exact test.

**Table 3:** Most frequent diagnostics at the RCP virtual clinic.

Diagnose	Origin			
	Triage program		Others	
	(*n* = 230)	%	(*n* = 423)	%
Normal	50	21,7%	57	13,5%
VSD	38	16,5%	80	18,9%
ASD	21	9,1%	45	10,6%
PDA	38	16,5%	19	4,5%
T4F	5	2,2%	17	4,0%
PVS	2	0,9%	16	3,8%
AVSD	4	1,7%	10	2,4%
Others	72	31,3%	179	42,3%

PDA: patent ductus arteriosus; VSD: Ventricular Septal Defect; ASD: Atrial Septal Defect; AVSD: Atrioventricular Septal Defect; T4F: Tetralogy of Fallot; PVS: Pulmonary Valve Stenosis.

## References

[1] Christianson A., Howson M., Modell B. (2006). *March of Dimes Global Report on Birth Defects. The Hidden Toll of Dying and Disabled Children*.

[2] Liu S., Liu J., Tang J., Ji J., Chen J., Liu C. (2009). Environmental risk factors for congenital heart disease in the Shandong Peninsula, China: a hospital-based case–control study. *Journal of Epidemiology*.

[3] Tandon A., Sengupta S. (2010). Factors for congenital heart sisease CHD in Vellore, India. *Current Research Journal of Biological Sciences*.

[4] Caneo L. F. (2012). Pediatric cardiovascular surgery: what we must preserve, what we should improve and what we must transform. *Brazilian Journal of Cardiovascular Surgery*.

[5] Pinto Júnior V. C., Rodrigues L. C., Muniz C. R. (2009). Reflexions about formulation of politics for attention to cardiovascular pediatrics in Brazil. *Brazilian Journal of Cardiovascular Surgery*.

[6] Moser L. R. D. N., Diogenes T. C. P., de Souza V. O. P., de Oliveira A. R. F., Mourato F. A., Mattos S. D. S. (2014). Novo modelo de teletriagem das cardiopatias congênitas. *Jornal Brasileiro de TeleSSaúde*.

[7] Moser L., Diogenes T., Mourato F. A., Mattos S. D. S. (2015). Learning echocardiography and changing realities through telemedicine. *Medical Education*.

[8] Ministério da Saúde do Brasil http://www2.datasus.gov.br/.

[9] Mattos S. S., Regis C. T., de Araújo J. S. S. (2013). Pediatric cardiology in public health: a tele-network covering over 55000 km^2^ of underserved regions in Brazil. *Cardiovascular Journal of Africa*.

[10] Rivera F. J. U., Artmann E. (2010). Planning and management in health: historical and tendencies based on a communicative view. *Ciência & Saúde Coletiva*.

[11] Miyague N. I., Cardoso S. M., Meyer F. (2003). Epidemiological study of congenital heart defects in children and adolescents: analysis of 4,538 cases. *Arquivos Brasileiros de Cardiologia*.

[12] Rivera I. R., da Silva M. A. M., Fernandes J. M. G., Thomaz A. C. P., Soriano C. F. R., de Souza M. G. B. (2007). Congenital heart diseases in the newborn: from the pediatrician's request to the cardiologist's evaluation. *Arquivos Brasileiros de Cardiologia*.

